# Lipid profile and prognosis in patients with coronary heart disease: a meta-analysis of prospective cohort studies

**DOI:** 10.1186/s12872-020-01835-0

**Published:** 2021-02-03

**Authors:** Xiangmei Zhao, Dongying Wang, Lijie Qin

**Affiliations:** 1 Department of Emergency, Henan Provincial People’s Hospital, People’s Hospital of Zhengzhou University, People’s Hospital of Henan University, No. 7 Weiwu Road, Zhengzhou, 450003 Henan China; 2 College of Food Science and Technology, Henan University of Technology, Zhengzhou, 450001 China

**Keywords:** Lipids, Prognosis, Coronary disease, Cardiovascular infections, Meta-analysis

## Abstract

**Background:**

This meta-analysis based on prospective cohort studies aimed to evaluate the associations of lipid profiles with the risk of major adverse cardiovascular outcomes in patients with coronary heart disease (CHD).

**Methods:**

The PubMed, Embase, and Cochrane Library electronic databases were systematically searched for prospective cohort study published through December 2019, and the pooled results were calculated using the random-effects model.

**Results:**

Twenty-one studies with a total of 76,221 patients with CHD met the inclusion criteria. The per standard deviation (SD) increase in triglyceride was associated with a reduced risk of major adverse cardiovascular events (MACE). Furthermore, the per SD increase in high-density lipoprotein cholesterol (HDL-C) was associated with a reduced risk of cardiac death, whereas patients with lower HDL-C were associated with an increased risk of MACE, all-cause mortality, and cardiac death. Finally, the risk of MACE was significantly increased in patients with CHD with high lipoprotein(a) levels.

**Conclusions:**

The results of this study suggested that lipid profile variables could predict major cardiovascular outcomes and all-cause mortality in patients with CHD.

## Background

Cardiovascular disease (CVD) is the leading cause of morbidity and mortality worldwide, accounting for nearly 30% of the total deaths based on the World Health Organization (WHO) statistics. The WHO reported that about 17.3 million people have died of CVD in 2016 and that this number will reach up to 23.3 million by 2030 [1]. Currently, pharmacological therapies including antiplatelet agents, angiotensin-converting enzyme inhibitors/angiotensin receptor blockers, beta-blockers and lipid-lowering drugs play a crucial role in the secondary prevention of CVD [2–4]. However, a residual CVD risk remains, for which further management needs to be identified.

Numerous studies have demonstrated the role of the lipid profile in the progression of CVD. Increases in triglyceride (TG) and total cholesterol (TC) levels could affect the constriction and abstraction of vessels in the heart, which are significantly correlated with the risk of CVD [5]. Moreover, increases in the low-density lipoprotein cholesterol (LDL-C) level could induce arteriosclerosis owing to accumulation of LDL-C in the intima-media of the artery, which could then promote thrombocytopoiesis [6]. However, the CVD risk might be reduced in persons with increased high-density lipoprotein cholesterol (HDL-C) levels. Therefore, individuals with high HDL-C and low non-HDL-C may be protected against the risk of CVD.

The ACC/AHA guideline used the intensity of statin therapy as the goal of treatment and recommend the maximum appropriate intensity of statin without adverse effects should be applied [7]. The ESC/EAS Guidelines suggested the treatment targets and goals for CVD prevention and the secondary targets of LDL-C were < 70 mg/dL, < 100 mg/dL, and < 115 mg/dL for very high-risk, high-risk, and low to moderate risk population, respectively [8]. The Japan Atherosclerosis Society Guidelines found the target for lipid profiles management in secondary preventing coronary artery diseases were < 100 mg/dL or < 70 mg/dL of LDL-C, < 130 mg/dL or < 100 mg/dL of non-HDL-C, < 150 mg/dL of TG, and > 40 mg/dL of HDL-C [9]. Although potential roles of lipid profile variables on the progression of CVD have been demonstrated; however, the impact of the lipid profile on the prognosis of patients with coronary heart disease (CHD) remains controversial. Clarifying the role of lipid profile variables in prognosis is particularly important in patients with CHD, as no systematic review and meta-analysis has provided definitive conclusions. Therefore, we attempted a large-scale examination of prospective cohort studies to determine the role of the lipid profile on the prognosis of patients with CHD.

## Methods

### Data sources, search strategy, and selection criteria

This review was conducted and reported according to the Preferred Reporting Items for Systematic Reviews and Meta-analysis Statement issued in 2009 [10]. The eligible studies for inclusion in the review were those with a prospective cohort design and that investigated the role of lipid profile variables on prognosis in patients with CHD. There were no restrictions with respect to publication language and status. We systematically searched the PubMed, Embase, and Cochrane Library electronic databases from their inception up to December 2019, using the following core search terms: (“Atherosclerosis” OR “Coronary Disease” OR “Coronary Artery Disease” OR “Coronary Occlusion” OR “Angina Pectoris”) AND (“total cholesterol” OR “triglyceride” OR “low-density lipoprotein” OR “high-density lipoprotein”) AND (“Death” OR “Recurrence” OR “Relapses” OR “Secondary Prevention” OR “risk” OR “prediction” OR “association” OR “correlation”) AND (“cohort” OR “prospective”). The detail of search strategy in PubMed are presented in Additional file 1. The reference lists of relevant review and original articles were also reviewed through manual searches to select any new eligible study.

Two authors independently performed the literature search and study selection following a standardized approach, and any inconsistencies between these 2 authors were resolved through a group discussion. The studies were judged for eligibility based on (1) study design (must be a prospective cohort study), (2) participants (all recruited patients must have a CHD diagnosis), (3) investigated variables (TC, TG, LDL-C, HDL-C, and lipoprotein(a)), (4) outcomes (major adverse cardiovascular events [MACE], all-cause mortality, and cardiac death), and (5) the investigated outcomes needed reported ≥ 2 cohorts. Studies with a retrospective observational design were excluded because of various confounding factors that could affect the results.

### Data collection and quality assessment

Data collection and quality assessment were conducted by 2 authors, and any disagreement was resolved by a third author by referring to the original works. The collected information from the retrieved studies included the first authors’ surname, publication year, country, sample size, age at baseline, percentage of men, disease status, follow-up duration, exposure, adjusted factors, and investigated outcomes. We selected the effect estimate that was maximally adjusted for potential confounders if a study reported several multivariable-adjusted effect estimates. Study quality was assessed using the Newcastle–Ottawa Scale, which was based on selection (4 items: 4 stars), comparability (1 item: 2 stars), and outcome (3 items: 3 stars). The “star system” for the assessment of each individual study ranged from 0 to 9 stars [11].

### Statistical analysis

The role of the lipid profile on the prognosis of patients with CHD was assessed based on the effect estimates and corresponding 95% confidence intervals (CIs) in each individual study. The summary relative risks (RRs) with 95% confidence intervals (CIs) were calculated using the random-effects model [12, 13]. The heterogeneity of studies was assessed using the I^2^ and Q statistics, and *P* < 0.10 was considered to indicate a significant heterogeneity [14, 15]. Sensitivity analyses were conducted for factors reported in ≥ 5 cohorts to assess the impact of a single study on the overall analysis [16]. Publication bias was assessed using funnel plots from Egger and Begg test results for factors reported in ≥ 5 cohorts [17, 18]. All reported *P* values are 2-sided, and *P* < 0.05 was considered to indicate statistical significance for all included studies. Statistical analyses were performed using STATA software (version 12.0; Stata Corporation, College Station, TX, USA).

## Results

### Literature search

**Fig. 1:**
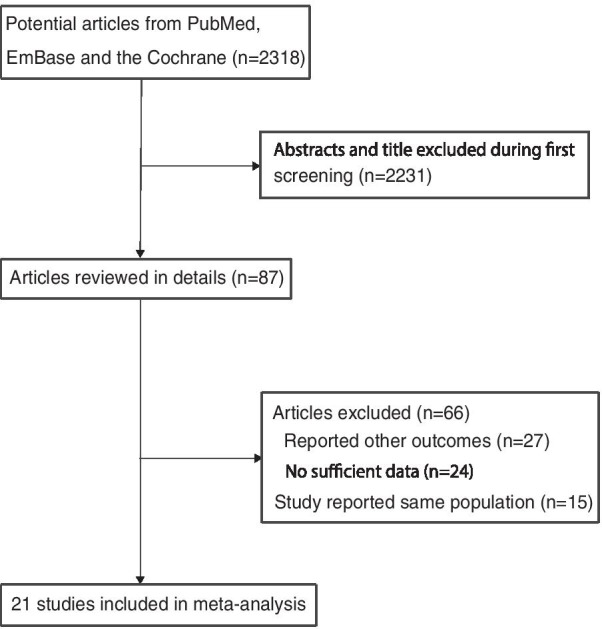
Flow diagram of the literature search and study selection

The electronic searches of PubMed, Embase, and the Cochrane Library yielded 2318 records, of which 2231 were excluded for being duplicates and for having irrelevant topics. A total of 87 studies were selected for further evaluation, and 21 prospective cohort studies with a total of 76,221 patients with CHD were selected for the final meta-analysis (Fig. 1) [19–39]. No new eligible study was detected by manual search of the reference lists of retrieved studies.

### Study characteristics

Table 1 summarizes the baseline characteristics of the included studies. A total of 21 studies published between 1995 and 2018 were analyzed, and each study included from 102 to 11,563 patients. Two studies included male patients, 1 study included female patients, while the remaining 18 studies included both male and female patients. The follow-up duration ranged from 1.0 to 10.3 years, and the study quality ranged from 6 to 8 stars. Twelve studies were conducted in Western countries, and the remaining 9 studies were conducted in Eastern countries.

**Table 1:** Baseline characteristics of the studies included in the systematic review and meta-analysis

Study	Publication year	Country	Sample size	Age at baseline	Percentage male (%)	Disease status	Follow-up (year)	Exposure	Adjusted factors	NOS score
Sigurdsson [19]	1995	Iceland	9141	34–79	100.0	CHD	5–20	Per SD increment	Age, smoking, glucose tolerance 90 min, heart volume	7
Tervahauta [20]	1995	Finland	171	65–84	100.0	CHD	5.0	Per SD increment	Age, area of residence, SBP, DBP, BMI, smoking habit and use of antihypertensive medication	6
Behar [21]	1997	Israel	11,563	45–74	78.3	CHD	3.3	Per SD increment; TC: < 160 mg/dL versus > 160 mg/dL	Age, gender, HDL; glucose, NYHA, previous MI, DM, COPD, hypertension, PVD, angina, current smoking	8
Mabuchi [22]	2002	Japan	4599	60.1	42.2	Hypercholesterolemia and CHD	6.0	Per 1 Log TG increment	Gender, age, hypertension, DM mellitus, smoking habit, and a history of MI	7
Vittinghoff [23]	2003	US	2763	66.6	0.0	CHD	4.1	Per SD increment	Age, ethnicity, smoking, alcohol, exercise, DM, previous MI, BMI, waist-to-hip ratio, hypertension, history of congestive heart failure	8
Leander [24]	2007	Sweden	1635	45–70	67.3	CHD	6–9	HDL (< 30.9/< 38.7 mg/dL versus > 30.9/ > 38.7 mg/dL)	Family history of CHD, current smoking, ex-smoking, job strain, PA, central obesity, DM, hypertension, hypercholesterolemia, low socio-economic status, high peak cardiac enzyme, heart failure, beta-blocker therapy, and thrombolysis	8
Al-Mallah [25]	2009	US	517	62.5	59.6	Non ST segment elevation MI	3.0	LDL (> 105 versus < 105 mg/dL)	Crude	6
Ghazzal [26]	2009	US	4088	64.8	70.9	CHD after PCI	1.0	HDL (< 35 versus > 35 mg/dL)	Age, DM, PCI, EF	7
Seo [27]	2011	Korea	2693	62.5	66.1	CHD after PCI	2.3	HDL (< 40/50 versus > 40/50 mg/dL)	Age, sex, hypertension, DM, chronic renal disease, current smoker, ACS, ejection fraction, baseline HDL, baseline LDL, baseline TG, baseline hs-CRP, follow-up LDL, follow-up TG, follow-up hs-CRP, total stent length, mean stent diameter, number of stent, number of B2/C lesions	7
Bacquer [28]	2013	Europe	5216	≤ 70.0	76.0	CHD	5.4	TC (< 174 versus > 232 mg/dL); HDL (< 38.7/46.4 versus > 46.4/54.1 mg/dL); LDL (> 116 versus < 96.7 mg/dL)	Age and gender	7
Lin [29]	2013	China	1114	65.5	74.9	CHD	5.3	HDL (< 40/45 versus > 40/45 mg/dL)	Age, gender, smoking status, LDL, uric acid, creatinine, hypertension, DM, stroke, cancer, number of blocked coronary artery, MI, PCI, CABG, and medication status	7
van de Woestijne [30]	2013	The Netherlands	5731	60.0	74.1	clinically manifest vascular disease	4.9	TG (198.4 versus < 85.9 mg/dL)	Age, gender, smoking, lipid-lowering medication, BMI and LDL-C	8
Ding [31]	2014	China	1916	63.7	65.2	CHD	3.1	HDL (40–49 versus > 70 mg/dL); LDL (> 190 versus < 70 mg/dL)	Age, gender, education, marriage, leisure-time physical activity, smoking, alcohol drinking, type, severity, duration, and treatment of CHD, history of DM, history of heart failure, BMI, SBP, glomerular filtration rate, and use of antihypertensive drugs, antidiabetic drugs, antiplatelet drugs, use of cholesterol-lowering drugs	7
Martin [32]	2014	US	4879	60.4	66.8	Acute MI	5.0	HDL (< 40 versus > 40 mg/dL)	GRACE score, age, sex, race, insurance, education, tobacco use, DM, hypertension, AUDIT alcohol use scores, PA, BMI, non-HDL-C, log-transformed triglycerides, statins, and non-statin lipid-modifying medications, and site	7
Park [33]	2015	Korea	595	63.5	65.2	CHD after PCI	3.0	Lipoprotein(a) (> 50 versus < 50 mg/dL)	Gender, age, diabetes mellitus, hypertension, hyperlipidemia, smoking, multivessel disease, minimal luminal diameter after PCI, reference vessel diameter after PCI, LDLcholesterol, total lesion length	6
Li [34]	2016	China	591	57.8	71.2	CHD	1.4	HDL (< 41.6 versus > 41.6 mg/dL)	Age, male, BMI, smoke, family history of CHD, previous PCI/CABG, previous MI, SBP, LDL, TG, and glucose	6
Liu [35]	2017	China	4205	57.7	71.4	CHD	2.3	Not mentioned	Age, gender, BMI, hypertension, DM, smoking, family history of CHD, left ventricular ejection fraction	7
Tsai [36]	2017	China	1520	69.0	72.8	CHD	2.7	Per SD increment	Age, gender, education, marital status, malnourished, economic situation, smoking, alcohol, PA, DM, hypertension, medications	7
Nordenskjöld [37]	2018	Sweden	9092	65.5	38.0	MI with with non-obstructive coronary artery disease	4.5	Per SD increment	Gender, age, DM, hypertension, smoking status, previous MI, previous stroke, ECG changes at admission, creatinine, CRP, total cholesterol, LVEF measured during index stay and serious non-cardiac diseases	8
Dai [38]	2018	China	4090	61.1	67.9	CHD	3.3	Per SD increment	Other traditional cardiovascular risk factors	7
Winter [39]	2018	Austria	102	37.3	79.4	Premature MI	10.3	Per SD increment	Age and gender	6

*ACS* acute coronary syndrome, *BMI* body mass index, *CABG* coronary artery bypass grafting, *CHD* coronary heart disease, *COPD* chronic obstructive pulmonary disease, *DBP* diastolic blood pressure, *DM* diabetes mellitus, *HDL* high density lipoprotein, *hs-CRP* high sensitivity C reactive protein, *LDL* low-density lipoprotein, *MI* myocardial infarction, *PA* physical inactivity, *PCI* percutaneous coronary intervention, *PVD* peripheral vascular disease, *SBP* systolic blood pressure, *TG* triglycerides

### Total cholesterol

**Fig. 2:**
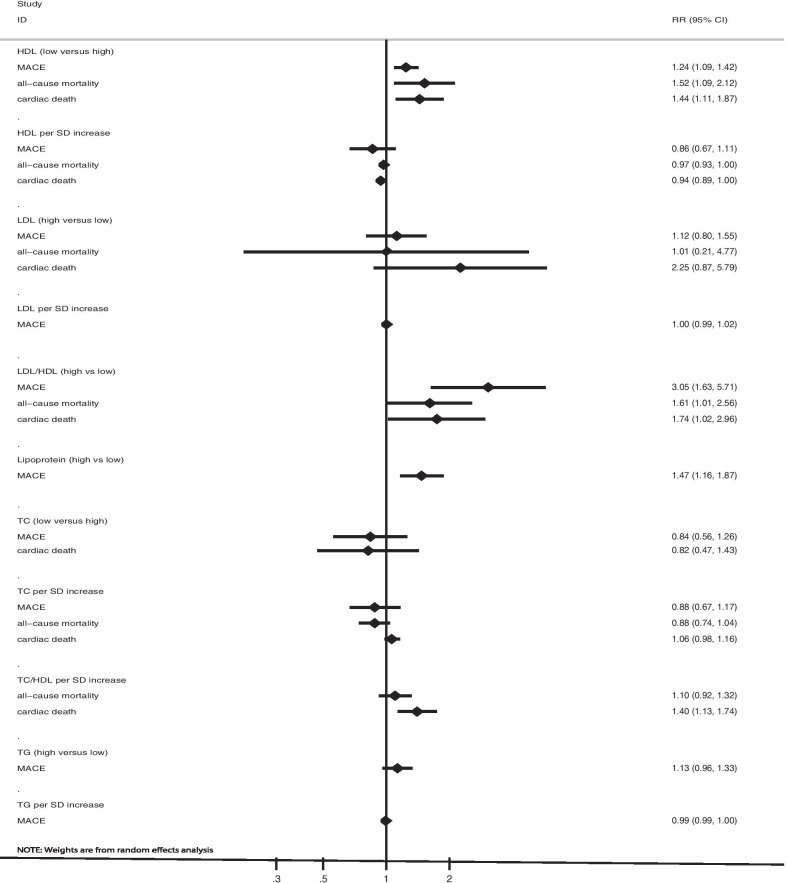
Summarized results with respect to the role of lipid profile variables on the risk of major cardiovascular events, all-cause mortality, and cardiac death in patients with coronary heart disease

The number of studies (cohorts) available for the analysis of the association of each outcome with the per standard deviation (SD) increase in TC was 5, 2, and 3 for MACE, all-cause mortality, and cardiac death, respectively (Fig. 2 and Table 2). Overall, we observed that the per SD increase in TC was not associated with the risk of MACE (RR: 0.88; 95% CI 0.67–1.17; *P* = 0.380; significant heterogeneity), all-cause mortality (RR: 0.88; 95% CI 0.74–1.04; *P* = 0.131; moderate heterogeneity), and cardiac death (RR: 1.06; 95% CI 0.98–1.16; *P* = 0.150; significant heterogeneity). The role of the per SD increase in TC on the risk of MACE in patients with CHD was altered when the study by Winter et al. [39], which had a longer follow-up duration, was excluded (Additional file 2). No significant publication bias was observed for MACE (Additional file 3).

**Table 2:** Summary results of lipid profile values and prognosis in patients with coronary heart disease

Factors	Outcomes	References	RR and 95% CI	*P* value	Heterogeneity (%)	*P* value for heterogeneity
TC per SD increase	MACE	[36–39]	0.88 (0.67–1.17)	0.380	67.4	0.015
	All-cause mortality	[20, 37]	0.88 (0.74–1.04)	0.131	50.6	0.155
	Cardiac death	[19–21]	1.06 (0.98–1.16)	0.150	79.7	0.007
TC (low versus high)	MACE	[21, 29]	0.84 (0.56–1.26)	0.389	56.6	0.129
	Cardiac death	[21, 28]	0.82 (0.47–1.43)	0.486	79.2	0.028
TG per SD increase	MACE	[36, 38, 39]	0.99 (0.99–1.00)	0.004	41.4	0.163
TG (high versus low)	MACE	[21, 30, 35]	1.13 (0.96–1.33)	0.134	22.5	0.275
LDL-C per SD increase	MACE	[23, 36, 38, 39]	1.00 (0.99–1.02)	0.640	81.4	< 0.001
LDL-C (high versus low)	MACE	[21, 25, 35]	1.12 (0.80–1.55)	0.512	69.7	0.037
	All-cause mortality	[25, 31]	1.01 (0.21–4.77)	0.994	84.1	0.012
	Cardiac death	[28, 31]	2.25 (0.87–5.79)	0.093	59.6	0.116
HDL-C per SD increase	MACE	[23, 36, 38, 39]	0.86 (0.67–1.11)	0.252	76.5	0.002
	All-cause mortality	[20, 21, 29]	0.97 (0.93–1.00)	0.065	85.2	< 0.001
	Cardiac death	[20, 21, 29]	0.94 (0.89–1.00)	0.048	89.2	< 0.001
HDL-C (low versus high)	MACE	[21, 24, 27, 32, 34, 35]	1.24 (1.09–1.42)	0.002	0.0	0.462
	All-cause mortality	[26, 27, 29, 31]	1.52 (1.09–2.12)	0.014	64.0	0.025
	Cardiac death	[27–29, 31]	1.44 (1.11–1.87)	0.006	29.8	0.223
Lipoprotein(a) (high vs low)	MACE	[23, 33, 39]	1.47 (1.16–1.87)	0.001	0.0	0.386

The number of studies (cohorts) available for the analysis of the association of each outcome with low versus high TC was 2, and 2 for MACE, and cardiac death, respectively (Fig. 2 and Table 2). Overall, we noted no significant associations of TC with the risk of MACE (RR: 0.84; 95% CI 0.56–1.26; *P* = 0.389; moderate heterogeneity) and cardiac death (RR: 0.82; 95% CI 0.47–1.43; *P* = 0.486; significant heterogeneity).

### Triglyceride

Data for the association of per SD increase in TG with the risk of MACE was available in 4 studies (cohorts) (Fig. 2 and Table 2). Overall, we noted that the per SD increase in TG was associated with a reduced risk of MACE (RR: 0.99; 95% CI 0.99–1.00; *P* = 0.004; moderate heterogeneity). Moreover, high TG was not associated with the risk of MACE (RR: 1.13; 95% CI 0.96–1.33; *P* = 0.134; unimportant heterogeneity).

### Low-density lipoprotein cholesterol

Data for the association of per SD increase in LDL-C with the risk of MACE was available in 5 studies (cohorts) (Fig. 2 and Table 2). There was no significant association between the per SD increase in LDL-C and the risk of MACE (RR: 1.00; 95% CI 0.99–1.02; *P* = 0.640; significant heterogeneity). The sensitivity analysis indicated that the risk of MACE was stable and not altered by the sequential exclusion of individual studies (Additional file 2). Moreover, no significant publication bias was detected for MACE (Additional file 3).

The number of studies (cohorts) available for the analysis of the association of each outcome with high versus low LDL-C was 3, 2, and 2 for MACE, all-cause mortality and cardiac death, respectively (Fig. 2 and Table 2). Overall, we observed that high LDL-C was not associated with the risk of MACE (RR: 1.12; 95% CI 0.80–1.55; *P* = 0.512; significant heterogeneity), all-cause mortality (RR: 1.01; 95% CI 0.21–4.77; *P* = 0.994; significant heterogeneity), and cardiac death (RR: 2.25; 95% CI 0.87–5.79; *P* = 0.093; moderate heterogeneity).

### High-density lipoprotein cholesterol

The number of studies (cohorts) available for the analysis of the association of each outcome with the per SD increase in HDL-C was 5, 4, and 4 for MACE, all-cause mortality, and cardiac death, respectively (Fig. 2 and Table 2). We observed that the per SD increase in HDL-C was associated with a reduced risk of cardiac death (RR: 0.94; 95% CI 0.89–1.00; *P* = 0.048; significant heterogeneity), whereas it was not associated with the risk of MACE (RR: 0.86; 95% CI 0.67–1.11; *P* = 0.252; significant heterogeneity) and all-cause mortality (RR: 0.97; 95% CI 0.93–1.00; *P* = 0.065; significant heterogeneity). The sensitivity analysis indicated that the per SD increase in HDL-C might produce a protective effect against MACE (Additional file 2). No significant publication bias was observed (Additional file 3).

The number of studies (cohorts) available for the analysis of the association of each outcome with low versus high HDL-C was 7, 5, and 5 for MACE, all-cause mortality, and cardiac death, respectively (Fig. 2 and Table 2). Overall, low HDL-C produced an excess risk of MACE (RR: 1.24; 95% CI 1.09–1.42; *P* = 0.002; with no evidence of heterogeneity), all-cause mortality (RR: 1.52; 95% CI 1.09–2.12; *P* = 0.014; significant heterogeneity), and cardiac death (RR: 1.44; 95% CI 1.11–1.87; *P* = 0.006; unimportant heterogeneity). The pooled results for MACE, all-cause mortality, and cardiac death varied after excluding individual studies, owing to marginal 95% CI (Additional file 2). No significant publication bias was detected for MACE, all-cause mortality, and cardiac death (Additional file 3).

### Lipoprotein(a)

Data for the association of high versus low lipoprotein(a) with the risk of MACE was available in 3 studies (cohorts) (Fig. 2 and Table 2). The summary RR indicated that the risk of MACE was significantly increased in patients with CHD with high lipoprotein(a) (RR: 1.47; 95% CI 1.16–1.87; *P* = 0.001; with no evidence of heterogeneity).

## Discussion

This systematic review and meta-analysis of prospective studies evaluated the role of the lipid profile on the risk of MACE, all-cause mortality, and cardiac death in patients with CHD. This comprehensive quantitative study included a total of 76,221 patients with CHD from 21 prospective cohort studies with a wide range of patient characteristics. The results suggested that in patients with CHD, increased TG was associated with a reduced risk of MACE. Moreover, low HDL-C was associated with an increased risk of MACE, all-cause mortality, and cardiac death. Finally, high lipoprotein(a) was associated with an increased risk of MACE in patients with CHD.

No previous systematic review and meta-analysis has focused on this topic, although numerous studies have illustrated the effects of lipid profile management in the secondary prevention of major cardiovascular outcomes. Gutierrez et al. conducted a meta-analysis of 11 randomized controlled trials and found that the use of statin for lipid profile management significantly reduced the risk of cardiovascular events in both sexes, whereas statin therapy had no significant effect on the risk of stroke and all-cause mortality in women [40]. Navarese et al. conducted a meta-analysis of 34 trials and found more intensive versus less intensive LDL-C lowering could further reduction in risk of total and cardiovascular mortality for patients with higher baseline LDL-C levels [41]. However, they did not focused on CHD patients. A meta-analysis including 5 studies with 4351 diabetic patients with manifest CVD was conducted by de Vries et al. The authors pointed out that both intensive and standard-dose statin therapy could produce a significant reduction in the risk of any major cardiovascular or cerebrovascular event [42]. However, most patients with CHD routinely use lipid management agents, and whether the lipid profile should be monitored in these patients remains controversial. Afilalo et al. conducted a meta-analysis of 6 trials and found intensive statin therapy was associated with a reduction in MACE and admission to hospital for heart failure as compared with moderate statin therapy. Moreover, they point out intensive statin therapy significantly reduced all-cause mortality in patients with recent acute coronary syndrome, while this effect was not observed for patients with stable CHD [43]. A meta-analysis conducted by Yan et al. found intensive statin therapy could further reduction the recurrent risk of MACE [44]. The results of previous studies mainly focused on the reduction in LDL-C, and the potential role of other lipid profiles on the prognosis of CHD remains unclear. Moreover, the long-term event monitoring study found DM, hypertension, TG, and LDL-C should be controlled for patients treated with statin to avoid further vascular events [45]. Therefore, the current comprehensive quantitative meta-analysis was conducted to evaluate the role of the lipid profile on the prognosis of patients with CHD.

The current study indicated that in patients with CHD, low TC was not associated with the risk of MACE and cardiac death. The potential reason for this result could be the twice higher prevalence of noncardiac death in the low TC group and cancer being the most frequent cause of noncardiac death. Moreover, we observed that patients with increased TG and LDL-C was not associated with the risk of MACE, all-cause mortality, and cardiac death. The result for TG was based on another study [46] that included the same population as that in the study by Behar et al. [21]. The authors pointed out that the association risk was balanced after adjusting for other risk factors and comorbidities [46].

The summary results indicated that CHD patients with low HDL-C have an excess risk of MACE, all-cause mortality, and cardiac death. Several included studies reported consistent results. Seo et al. found that patients with low HDL-C had a significantly increased risk of MACE after 832 days of follow-up, whereas low HDL-C had no significant impact on all-cause mortality and cardiac death [27]. The potential reason for this could be the shorter duration of follow-up than what was needed to show a clinical benefit, especially for the lower-than-expected all-cause mortality and cardiac death rates, which always yielded broad confidence intervals (i.e., no statistically significant difference). Ghazzal et al. found that low serum HDL-C was an independent risk factor for 1-year mortality, and used 35 mg/dL as a cutoff value for defining high and low HDL-C levels [26]. De Bacquer et al. suggested that the risk of cardiac death was significantly increased in patients with CHD with low HDL-C [28]. Lin et al. indicated that low HDL-C level did not affect the risk of all-cause mortality and cardiac death in patients with CHD with a body mass index of > 25.0 kg/m^2^, whereas an increased risk of all-cause mortality and cardiac death was noted with low HDL-C level when the body mass index was < 25.0 kg/m^2^ [29]. Ding et al. found that the risk of all-cause mortality and cardiac death had a U-shaped correlation with HDL-C after adjusting for major CVD risk factors [31]. They pointed out that the antiatherogenic effect of HDL-C could reverse macrophage cholesterol transport, which, in turn, could stimulate nitric oxide production, inhibit endothelial apoptosis, and induce endothelial homeostasis [47, 48].

This study showed that the risk of MACE was significantly increased in CHD patients with high lipoprotein(a), which was consistent with the result of a previous study that found that the reduction in lipoprotein(a) was independently correlated with a reduced risk of MACE [49]. Furthermore, lipoprotein(a) level was not associated with the risk of all-cause mortality and cardiac death [33]. Finally, we noted LDL-C was not associated with the risk of MACE, all-cause mortality, and cardiac death in CHD patients. The potential reason for this could be CHD patients with strictly lipid profile management strategies to prevent the progression of major adverse cardiovascular outcomes.

The limitations of this meta-analysis are as follows: (1) the cutoff values of lipid profile variables varied among the included studies, which could affect the effect size of the risk of MACE, all-cause mortality, and cardiac death in patients with CHD; (2) the dose–response analysis were not conducted owing to it requires that the distributions of cases and persons or person-years and effect estimate (RRs or HRs) with the variance estimates for at least 3 quantitative exposure categories; (3) several outcomes were reported in only a few studies, and stratified analyses according to patients’ characteristics were not described; (4) heterogeneity among included studies were substantial, which not fully interpret by using a sensitivity analyses. These results could introduce by various disease status, background therapies, cutoff value of lipid profiles, and adjusted factors; (5) the adjusted factors were different among the included studies, which could affect the prognosis of patients with CHD; (6) unpublished data were not identified, which might cause an overestimation of the summary effect estimate; and (7) the role of apolipoprotein in patients with CHD was not investigated in the included studies. Further studies investigating any potential role of apolipoprotein on the progression of major cardiovascular outcomes in patients with CHD are needed.

## Conclusions

The results of this study suggested that the lipid profile could affect the progression of MACE, all-cause mortality, and cardiac death in patients with CHD. Further large-scale prospective studies should be conducted with a focus on patients with specific characteristics to investigate the secondary prevention of major cardiovascular outcomes and mortality.

## Supplementary Information

**Additional file 1**. Search strategy in PubMed.

**Additional file 2**. Sensitivity analysis.

**Additional file 3**. Funnel plot.

## Data Availability

All data generated or analysed during this study are included in this published article [and its supplementary information files].

## Abbreviations

CHD: Coronary heart disease
CIs: Confidence intervals
CVD: Cardiovascular disease
HDL: High-density lipoprotein
LDL: Low-density lipoprotein
MACE: Major adverse cardiovascular events
SD: Standard deviation
TC: Total cholesterol
TG: Triglyceride
WHO: World Health Organization
