# Supplementary material for: Lipid profile and prognosis in patients with coronary heart disease: a meta-analysis of prospective cohort studies
Source: BMC Cardiovasc Disord. 2021 Feb 3;21:69. doi: 10.1186/s12872-020-01835-0 (PMC7860615; doi:10.1186/s12872-020-01835-0)
Supplement: Supplementary file 1 — Additional file 1. Search strategy in PubMed. [file 12872_2020_1835_MOESM1_ESM.docx]

**Search strategy in PubMed**

("Atherosclerosis"[Mesh] OR "Atherosclerotic cardiovascular disease"[Title/Abstract] OR ASCVD[Title/Abstract] OR "Coronary Disease"[Mesh] OR "Coronary Disease"[Title/Abstract] OR "Coronary Artery Disease"[Mesh] OR "Coronary Artery Disease"[Title/Abstract] OR "Coronary Occlusion"[Mesh] OR "Coronary Occlusion"[Title/Abstract] OR "Angina Pectoris"[Mesh] OR "Angina Pectoris"[Title/Abstract]) AND ("total cholesterol"[Mesh] OR "total cholesterol"[Title/Abstract] OR "triglyceride" [Mesh] OR "triglyceride"[Title/Abstract] OR "low density lipoprotein"[Mesh] OR "low density lipoprotein"[Title/Abstract] OR "high density lipoprotein"[Mesh] OR "high density lipoprotein"[Title/Abstract]) AND ("Death"[Mesh] OR death[Title/Abstract] OR "Recurrence"[Mesh] OR recurren*[Title/Abstract] OR Relapses*[Title/Abstract] OR "Secondary Prevention"[Mesh] OR secondary prevention[Title/Abstract] OR risk OR prediction[Title/Abstract] OR association[Title/Abstract] OR correlation[Title/Abstract]) AND ("cohort" OR "prospective")
