# Supplementary material for: Lipid profile and prognosis in patients with coronary heart disease: a meta-analysis of prospective cohort studies
Source: BMC Cardiovasc Disord. 2021 Feb 3;21:69. doi: 10.1186/s12872-020-01835-0 (PMC7860615; doi:10.1186/s12872-020-01835-0)
Supplement: Supplementary file 2 — Additional file 2. Sensitivity analysis. [file 12872_2020_1835_MOESM2_ESM.docx]

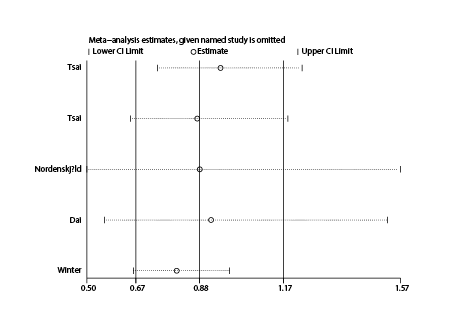


Figure S1. Sensitivity analysis for per SD increase in TC on the risk of MACE in CHD patients


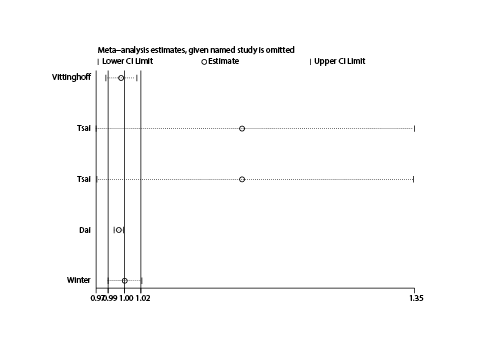


Figure S2. Sensitivity analysis for per SD increase in LDL on the risk of MACE in CHD patients


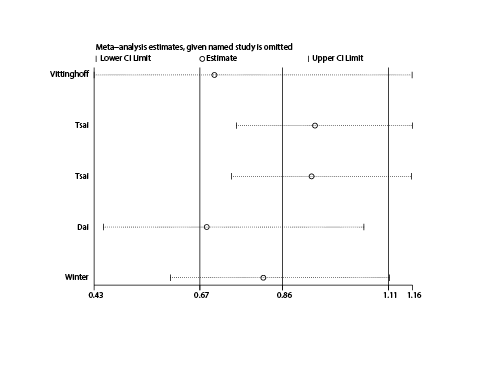


Figure S3. Sensitivity analysis for per SD increase in HDL on the risk of MACE in CHD patients


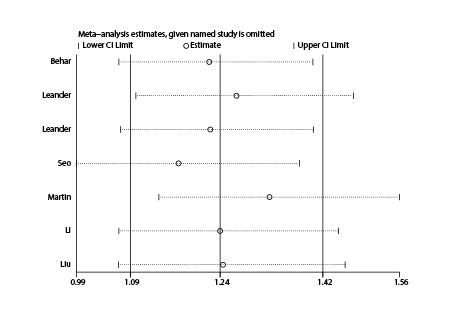


Figure S4. Sensitivity analysis for low versus high HDL on the risk of MACE in CHD patients


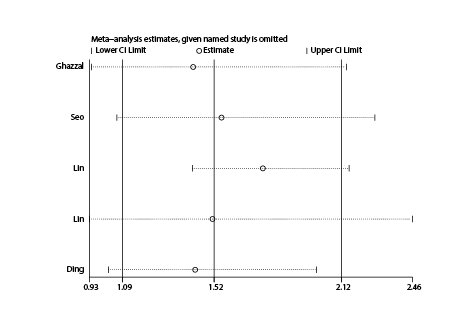


Figure S5. Sensitivity analysis for low versus high HDL on the risk of all-cause mortality in CHD patients


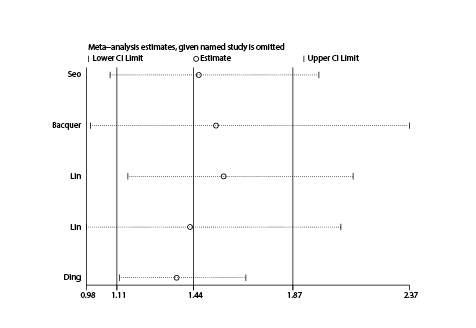


Figure S6. Sensitivity analysis for low versus high HDL on the risk of cardiac death in CHD patients
