# Supplementary material for: Lipid profile and prognosis in patients with coronary heart disease: a meta-analysis of prospective cohort studies
Source: BMC Cardiovasc Disord. 2021 Feb 3;21:69. doi: 10.1186/s12872-020-01835-0 (PMC7860615; doi:10.1186/s12872-020-01835-0)
Supplement: Supplementary file 3 — Additional file 3. Funnel plot. [file 12872_2020_1835_MOESM3_ESM.docx]

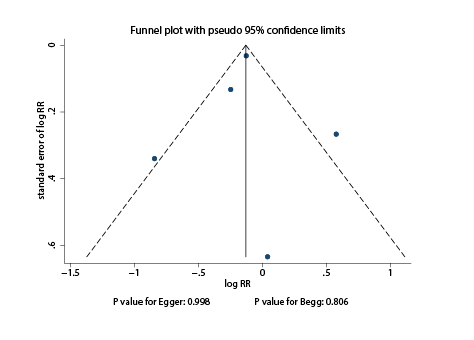


Figure S1. Funnel plot for per SD increase in TC on the risk of MACE in CHD patients


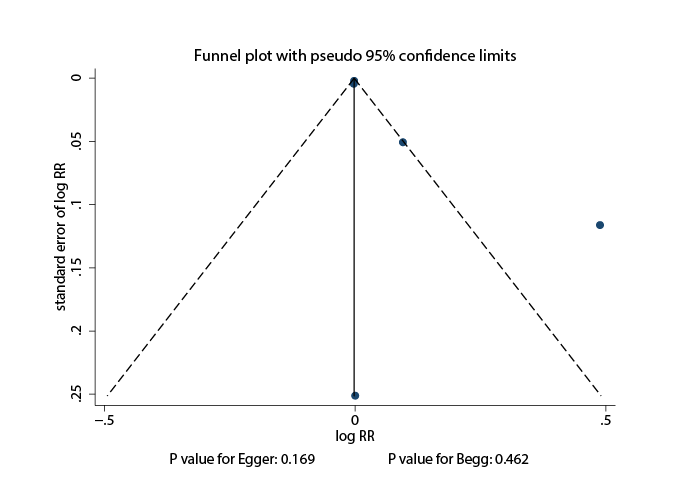


Figure S2. Funnel plot for per SD increase in LDL on the risk of MACE in CHD patients


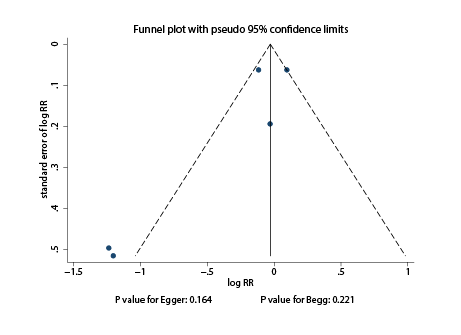


Figure S3. Funnel plot for per SD increase in HDL on the risk of MACE in CHD patients


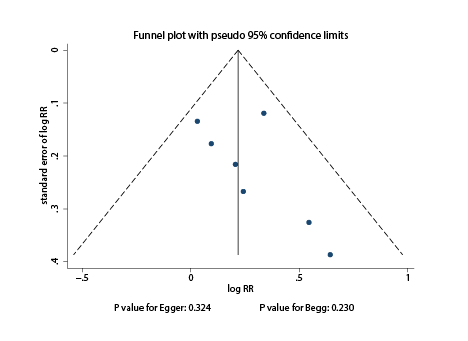


Figure S4. Funnel plot for low versus high HDL on the risk of MACE in CHD patients


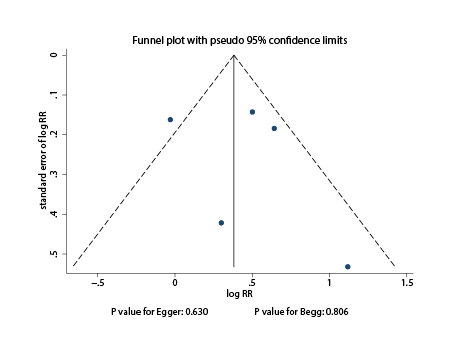


Figure S5. Funnel plot for low versus high HDL on the risk of all-cause mortality in CHD patients


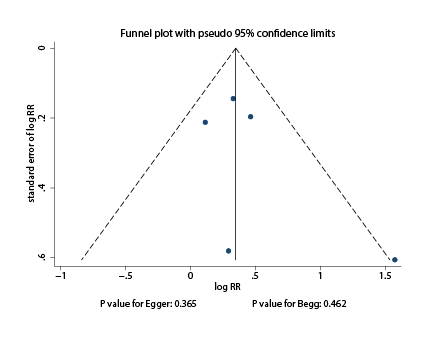


Figure S6. Funnel plot for low versus high HDL on the risk of cardiac death in CHD patients
